# Artificial intelligence in thyroid ultrasound

**DOI:** 10.3389/fonc.2023.1060702

**Published:** 2023-05-12

**Authors:** Chun-Li Cao, Qiao-Li Li, Jin Tong, Li-Nan Shi, Wen-Xiao Li, Ya Xu, Jing Cheng, Ting-Ting Du, Jun Li, Xin-Wu Cui

**Affiliations:** ^1^ Department of Ultrasound, The First Affiliated Hospital of Shihezi University, Shihezi, China; ^2^ NHC Key Laboratory of Prevention and Treatment of Central Asia High Incidence Diseases, First Affiliated Hospital, School of Medicine, Shihezi University, Shihezi, China; ^3^ Department of Medical Ultrasound, Tongji Hospital, Tongji Medical College, Huazhong University of Science and Technology, Wuhan, Hubei, China

**Keywords:** artificial intelligence, thyroid, ultrasound, machine learning, deep learning

## Abstract

Artificial intelligence (AI), particularly deep learning (DL) algorithms, has demonstrated remarkable progress in image-recognition tasks, enabling the automatic quantitative assessment of complex medical images with increased accuracy and efficiency. AI is widely used and is becoming increasingly popular in the field of ultrasound. The rising incidence of thyroid cancer and the workload of physicians have driven the need to utilize AI to efficiently process thyroid ultrasound images. Therefore, leveraging AI in thyroid cancer ultrasound screening and diagnosis cannot only help radiologists achieve more accurate and efficient imaging diagnosis but also reduce their workload. In this paper, we aim to present a comprehensive overview of the technical knowledge of AI with a focus on traditional machine learning (ML) algorithms and DL algorithms. We will also discuss their clinical applications in the ultrasound imaging of thyroid diseases, particularly in differentiating between benign and malignant nodules and predicting cervical lymph node metastasis in thyroid cancer. Finally, we will conclude that AI technology holds great promise for improving the accuracy of thyroid disease ultrasound diagnosis and discuss the potential prospects of AI in this field.

## 1 Introduction

With the rapid progress of modern medicine, especially the continuous development of imaging technology, the detection rate of thyroid diseases and thyroid cancer has shown a rapid growth trend both domestically and internationally (1, 2). Various auxiliary examination methods, such as ultrasound, computed tomography, and radioisotope scanning, are used to evaluate thyroid diseases (3). Among them methods, ultrasound has become the primary means of thyroid examination and diagnosis because of its advantages, such as convenience, real-time imaging display, non-radiation, and good tolerance (4, 5). However, the accurate identification of ultrasound images is highly related to physician’s experience, and the differences between different observers can be significant. Therefore, inexperienced physicians are at greater risk of misdiagnosis, underestimating the condition, or unnecessarily performing fine-needle aspiration (FNA) biopsies (6, 7). Therefore, taking advantage of artificial intelligence (AI) in thyroid disease ultrasound screening and diagnosis not only assist the radiologists in achieving more accurate imaging diagnosis with higher efficiency, but also lessen the radiologists’ workload (8).

AI, a branch of computer science that encompasses both machine learning (ML) algorithms and deep learning (DL) algorithms, is gaining increasingly popularity in the field of medicine. Due to its ability to process pixel values and derive insights from images, AI techniques are particularly well-suited for fields that rely on imaging data, such as gastroenterology (9), ophthalmology (10), dermatology (11), pathology (12), radiology (13), and ultrasonography (14). The exponential growth in the volume of medical data over the past decade has spurred the development of AI, which can automatically analyze complex medical images and provide more accurate and efficient diagnoses. By leveraging AI for thyroid disease ultrasound screening and diagnosis, radiologists can reduce their workload and improve the accuracy of their diagnoses.

Several scholars have examined the application of ultrasound AI in thyroid diseases thus far. However, most review studies focus on specific topics, such as distinguishing benign or malignant thyroid nodules or predicting cervical lymph node metastasis using ultrasound features. As a result, there is a need for a comprehensive review of the current state and future possibilities of AI in thyroid ultrasound. This article aims to provide a comprehensive review by discussing the fundamental theoretical knowledge of AI, including traditional ML and DL algorithms, and their clinical application in ultrasonic imaging of thyroid diseases, such as thyroid disease detection, thyroid segmentation, and differential diagnosis of thyroid nodules. Finally, this article addresses the challenges and prospects of AI in the clinical application of thyroid ultrasound.

## 2 Basic theoretical knowledge of AI

### 2.1 Conventional ML algorithms

Traditional ML algorithms typically rely on the pre-defined engineered features that accurately describe the regular patterns inherent in data extracted from regions of interest (ROI) with explicit parameters on the basis of expert knowledge. In the medical field, common ML algorithms such as support vector machines (SVM), Bayesian classifiers, et al. rely heavily on these predefined features (15, 16). While these features are considered to be discriminative, conventional ML algorithms are limited by their dependence on expert-defined features and cannot adapt to changes in different imaging methods or variations in signal-to-noise ratios.

### 2.2 DL algorithms

Unlike traditional ML algorithms, DL algorithms do not require predetermined features and regions of interest set by humans. Instead, they can automatically learn representations of information and gain experience from raw data (17). DL algorithms are composed of simple and nonlinear modules that are particularly effective at extracting features from ultrasound images (18). Various DL architectures have been explored to solve problems, with the convolutional neural network (CNN) being the most commonly utilized architecture (19).

**Figure 1:**
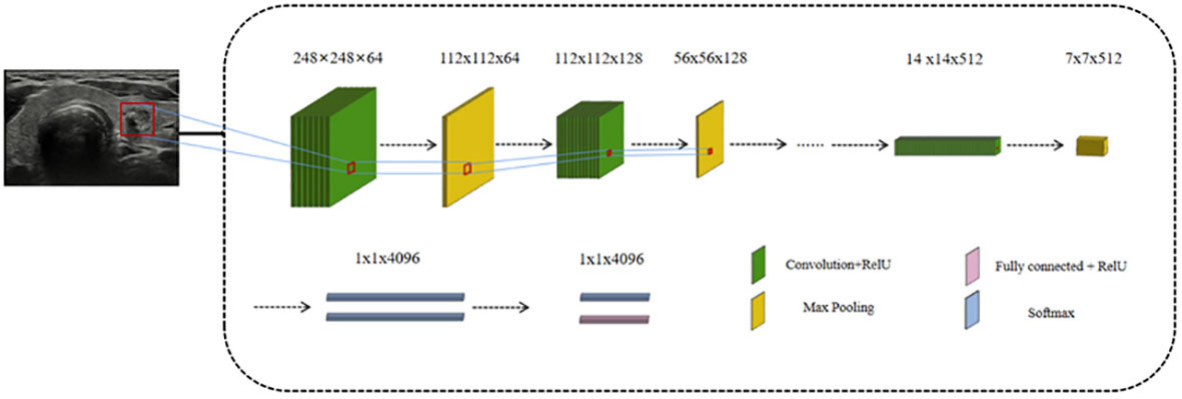
A typical convolutional neural network model.

In the 1990s, the use of CNNs expanded to include image processing (20). Compared to other approaches, CNNs utilize spatial and structural information more effectively. The network can directly input the original image, eliminating the need for preprocessing and complex feature extraction procedures that can lead to errors and classification biases. The structure of the convolutional neural network CNN generally includes the following layers: input layer, convolutional layer, pooling layer, fully linked layer and output layer (**Figure 1**). These layers map the input image information to the critical endpoint in turn through mapping, and learn more advanced image functions at the same time. The convolutional layer is the essential component of a CNN, responsible for extracting features from input images. Through data sharing between the input and output feature maps, the convolutional layer reduces the number of trainable parameters and overall model complexity, thereby facilitating network training. The initial convolutional layer typically extracts basic features, while subsequent layers iteratively extract increasingly complex content from lower-level functions (21). Pooling layers are periodically inserted between consecutive convolutional layers for feature extraction and information filtering. Its function is to decrease the dimension of each feature graph, reduce computing resources and control overfitting effectively while improving the fault tolerance of the model. The operations performed by the pooling layer are usually of the following types: maximum pooling, mean pooling, random pooling, gaussian pooling and training pooling, of which maximum pooling is the most common method (22). When a linear classifier is employed in the classification layer, maximum pooling has better classification performance than average pooling due to its better classification performance (23). The fully connected layer in a CNN serves as the classifier for the entire network, responsible for categorizing the extracted features. It combines the local information with feature identification from the convolution and pooling layers, refitting the extracted features and reducing loss of information. Due to its numerous connection weights, overfitting is a potential risk with the fully connected layer. To mitigate this risk, sparse connections and dropout methods have been suggested (24).

## 3 Application of AI in thyroid ultrasound

### 3.1 Detection of thyroid disease

Thyroid nodule images obtained through ultrasound are often distorted by echo disturbance and speckle noise. Thus, accurate recognition of these images typically requires the expertise of experienced physicians. However, DL algorithms that incorporate multiple image patterns have become increasingly prevalent in detecting thyroid lesions (25–28). As an automated method, a computer-aided detection system (CAD) can recognize and process predefined features, which is used in clinical practice. The combination of CAD and ultrasound in thyroid ultrasound image detection, compared with visual assessment, this method is helpful to find the pathological features that cannot be recognized by the naked eye, so as to improve the detection rate of thyroid lesions.

Ma et al. (25) performed a cascaded CNN to detect thyroid nodules automatically, utilizing 21532 images from 5842 patients. The model was designed to bypass potential errors that might arise during preprocessing, leading to inaccurate results and classification bias due to the feature set’s lack of robustness. And the result demonstrated that the model performs good detection efficiency with an area under the summary receiver operating characteristic curve (AUROC) of 98.51%. Another study by Li et al. (26) developed a papillary thyroid cancer detection model based on R-CNN, which demonstrated a sensitivity of 93.5%. Liu et al. (27) also employed the multi-scale detection network to automatically detect thyroid nodules with an accuracy rate of 97.5%. Acharya et al. (28) proposed a CAD system called ThyroScan, which utilizes seven significant wavelet features extracted from thyroid images of 232 normal thyroid and 294 Hashimoto’s thyroiditis patients. The fuzzy classifier showed an accuracy of 85% in detecting Hashimoto’s thyroiditis.

### 3.2 Segmentation: to achieve the segmentation of the precise boundary of the lesion

Thyroid ultrasound image segmentation, as one of the most commonly used image preprocessing methods, is usually used to detect and diagnose nodules and to estimate the volume. It is an essential part of CAD systems and the diagnosis of thyroid diseases (29). However, Raw thyroid ultrasound images contain inaccurate and incomplete information, leading to erroneous segmentation results. Therefore, precise segmentation of thyroid nodules is essential for accurate diagnosis of thyroid nodules. In addition, thyroid segmentation can be applied to estimate thyroid volume, which helps evaluate thyroid hormone secretion. Therefore, thyroid segmentation and thyroid nodule segmentation are essential links to improve the development of thyroid AI research, which is conducive to providing a reliable theoretical basis for radiologists’ diagnostic decisions.

Thyroid segmentation and nodule segmentation are classified into contour- and shape-based methods, region-based methods, machine and DL methods, and hybrid methods (30). Although these methods are used with different objectives, they share the same classification.

a) Contour- and shape-based methods: The method processes ultrasound images of the thyroid by obtaining information about the border or shape of the thyroid or thyroid nodule. Edge segmentation is a primary image segmentation method using a different edge detection operator (31). And the characteristic of contour segmentation is that the energy function is used as a measure of the coincidence between the prior model and the image data to make the contour curve approach the target contour (32). However, interfering with original image contrast and image artefacts, borders between thyroid nodules were sometimes discontinuous or false borders were detected. In addition, thyroid nodules are often irregular. Therefore, initial contour and prior shape information are usually required to improve the segmentation accuracy. b) Region-based method: This approach assumes that different regions in thyroid ultrasound images are inhomogeneous, thereby obtaining a minimized boundary energy function. However, when the differences between different regions are less significant, the application of this method is limited. c) Machine and DL methods: The method is based on a machine and DL algorithm to construct a classifier that can automatically extract features, and finally segment the target tissue area accurately, which increases the accuracy of classification. However, machine and DL classifiers require an amount of training data and take a long time to train (21). d) Hybrid methods: The above two or more methods are combined to achieve the purpose of improving the segmentation accuracy.

#### 3.2.1 Thyroid segmentation

In several studies, different automatic segmentation methods were used to segment thyroid ultrasound images with varying degrees of success. Poudel et al. (33) employed the graph cut (GC) method (34) to segment 1416 images, achieving a dice coefficient of 76.5%. Meanwhile, Narayan et al. (29) developed a method based on the principle of echo consistency to segment 52 thyroid images, resulting in a dice coefficient of 84.47%, higher than the average of 83.23% obtained by two experts. Chang et al. (35) developed a radial basis function neural network (RBFNN) approach to automatically segment thyroid gland in 3D. The segmentation method includes four steps thyroid region localization and image enhancement, feature extraction, training RBFNN and thyroid recovery. This study used 60 training patterns to train the RBFNN and evaluated the performance of the method by testing thyroid ultrasound images. The study found that the accuracy of the method is 96.52%. Selvathi et al. (36) developed support vector machines and extreme learning machines to develop an automatic segmentation method, resulting in segmentation accuracies of 84.78% and 93.56%, respectively. Poudel et al. (33) also proposed a 3D U-Net CNN model (37) for thyroid segmentation and compared its performance with four other methods(ACWE, GC, RF, and DT), achieving an average dice coefficient of 87.6%, which was higher than other methods.

#### 3.2.2 Thyroid nodule segmentation

Nugroho et al. (38) used bilateral filtering (39) to preprocess thyroid ultrasound images and then applied an active contour without edges (ACWE) model to segment thyroid nodules. The approach produced clearer nodule localizations and more accurate segmentation outcomes. However, the ACWE model assumes homogeneity of both foreground and background in the thyroid ultrasound image, leading to potential inaccuracies. To overcome the limitations of the ACWE model, Maroulis et al. (40) improved the ACWE model and proposed a variable background active contour (VBAC) model, which can mitigate the effects of inhomogeneous tissue in ultrasound images. The VBAC model was used to segment 71 thyroid nodule ultrasound images, and compared with the ACWE model. The VBAC model achieved a higher average overlap value of 91.1% compared to the ACWE model’s 84.8%, indicating its superior performance in nodule segmentation. Mylona et al. (41, 42) added Orientation Entropy (OE) based on the ACWE model to make the contour of the nodule closer to the target edge and leave the continuity of the contour unaffected. This study showed that the OE-ACWE model evolves faster than the ACWE model, and the average overlap rate of segmentation results was 83.70%. In addition, some scholars have studied the segmentation of thyroid nodules using DL methods. Ma et al. (43) proposed a CNN model to segment nodules in 22,123 thyroid ultrasound images with an average overlap of 86.83% using ten-fold cross-validation. Some scholars (44) have also proposed a cascaded convolutional neural network (CCNN) model for thyroid nodule segmentation, using 1000 images in the dataset and achieving an average overlap rate of 87.00% in the test set.

In addition, hybrid segmentation techniques have been investigated to improve segmentation models and automate the segmentation of solids. Zhou et al. (45) combined the GC model and the ACWE model to segment thyroid nodules. After research, it is concluded that the hybrid model performed better in segmentation and addressed boundary leakage issues more effectively. However, this study lacked quantitative results and could not yet be compared with other models. Legakis et al. (46) combined a maximum likelihood algorithm and an active contour model to segment nodules in thyroid ultrasound images. The study found that the average overlap rate of segmentation results is 92.30%.

Thyroid segmentation and thyroid nodule segmentation have become indispensable for modern medical ultrasound imaging diagnosis, aiding clinicians in making optimal diagnostic decisions. However, they do have certain limitations. Firstly, the current research on thyroid segmentation mainly focuses on the segmentation of normal thyroid, leaving the segmentation of an abnormal thyroid underexplored. The size and shape of abnormal thyroid tissue may present new challenges for thyroid segmentation. Secondly, most studies related to thyroid nodule segmentation aim to distinguish the nature of thyroid nodules but do not identify specific disease types.

### 3.3 Differentiation of malignant and benign thyroid nodules

#### 3.3.1 ML

Currently, some studies have combined the maximum likelihood algorithm with the analysis of ultrasonic image texture features for the differential diagnosis of thyroid nodules (**Table 1**). The main objective of most researchers is to evaluate the efficacy of ML algorithms in distinguishing between benign and malignant thyroid nodules. In this regard, CAD systems that utilize ML algorithms have become increasingly significant in assisting ultrasound imaging to enhance the precision of nodule assessment.

**Table 1:** Main results of ML algorithm in thyroid nodules ultrasound image studies.

Reference	Published year	Technique	No. of subjects	Features	Classifier	Main Performance
Hirning et al (47)	1989	US	55	Hist, GLCM	SVM	Accuracy:85.0%
Tsantis et al (16)	2005	US	120	Hist, GLCM, GLRLM	SVM	Accuracy:96.7%
Tsantis et al (48)	2009	US	85	Shape and size, Fractal, Wavelet	SVM, PNN	AUROC:0.96
Savelonas et al (46)	2009	US	171	His, shape and size, Fractal	SVM, k-NN	AUROC:0.95
Chang et al (49)	2010	US	61	GLCM, GLRLM; Wavelet	SVM	Accuracy:100%
Iakovidis et al (50)	2010	US	75	Hist, GLCM, FLBP	SVM	Accuracy:97.5%
Legakis et al (51)	2011	US	142	Textural, shape feature vectors	SVM	AUROC:0.93
Luo et al (52)	2011	Elastography	98	Waveform	LDA	Sensitivity:100%; Specificity:75.6%
Ding et al (53)	2011	Elastography	125	Hist, GLCM	SVM	Accuracy:93.6%; AUROC:0.97
Acharya et al (31)	2011	3D CEUS	20	GLCM, Wavelet	K-NN, PNN, DT	Sensitivity:98.0%; Specificity:99.8%; Accuracy:98.9%; AUROC:0.99
Acharya et al (54)	2012	3D HRUS	20	GLCM, Wavelet	AdaBoost	Sensitivity:100.0%; Specificity:100.0%; Accuracy:100.0%; AUROC:1.00
Acharya et al (55)	2012	3D CEUS	20	GLCM, Wavelet	K-NN, PNN, DT	Sensitivity:98.0%; Specificity:99.8%; Accuracy:98.9%
Acharya et al (56)	2012	3D HRUS 3D CEUS	20	FD, LBP, FS, LTE	SVM, DT, Sugeno Fuzzy, GMM, KNN, PNN, NB	HRUS Accuracy:100.0% CEUS Accuracy:98.1%
Acharya et al (57)	2013	3D CEUS	20	Wavelet	Fuzzy	Accuracy:99.1%
Zhu et al (58)	2013	US	689	na	ANN	Accuracy:83.1%; AUROC:0.83
Kim et al (59)	2015	US RTE	613	Hist, GLCM	na	AUROC:0.68
Song et al (60)	2015	US	155	GLCM	SVM, RT, RF, boost, logistic, ANN	AUROC:0.84
Ardakani et al (61)	2015	US	70	Hist, GLCM, GLRLM	1-NN, ANN	Sensitivity:94.5%; Specificity:100.0%; Accuracy:97.1%; AUROC:0.97
Ardakani et al (62)	2015	US	60	Wavelet	1-NN	Sensitivity:100.0%; Specificity:100.0%; Accuracy:100.0%; AUROC:1.00
Acharya et al (63)	2016	3D HRUS	242	Safe-Level SMOTE	SVM, KNN, MLP, C4.5decision tree	Accuracy:94.3%
Bhatia et al (64)	2016	SWE	105	GLCM	na	Sensitivity:97.5%; Specificity:90.0%; AUROC:0.97
Chang et al (65)	2016	US	59	Hist, intensity differences, elliptical fit, GLCM, GLRLM	SVM	AUROC:0.99
Wu et al (66)	2016	US	970	na	NB, SVM, RBF-NN	AUROC:0.91
Raghavendra et al (67)	2017	US	242	SGLD, Fractal	DT, LDA, QDA, NB, PNN, k-NN, SVM with different kernel functions	Sensitivity:90.3%; Specificity:98.6%; Accuracy:97.5%; AUROC:0.94
Yu et al (68)	2017	US	543	Hist, GLCM, GLRLM, NGLDM, Fractal	SVM, ANN	Sensitivity:100.0%; Specificity:87.9%; Accuracy:92.0%
Zhang et al (69)	2019	US RTE	2064	na	k-NN, RF, k-SVM, Logistic, LDA, CNN, adaptive boosting, NB, neural network	AUROC:0.94
Ouyang et al (70)	2019	US	1036	na	Ridge, Lasso-penalty, Elastic Net, RF, k-SVM, Neural Network	AUROC:0.95
Shin et al (71)	2020	US	348	GLCM, GLRLM, Gabor, and Haar wavelet	SVM, ANN	SVM: Accuracy:79.4% ANN: Accuracy:69.0%
Zhao et al (72)	2021	US SWE	743	contour, shape, textural phenotype, Hist, GLCM, GLRLM, GLSZM,NGTDM,GLDM,LTP,LDP,LBP, ect.	DT, NB, KNN, logistics regression, SVM, KNN-based bagging, RF, extremely randomized trees (xgboost), multi-layer perception, and gradient boosting tree	ML‐assisted SWE+US visual approach: AUC:0.951 SWE+US radiomics approach: AUC:0.834
Vijay et al (73)	2021	US	99	GLCM	ANN, SVM	Accuracy:96.0%
Matti et al (74)	2022	US	8339	Hist, GLCM	Random forest	AUC:0.75
M. Keutgen et al (75)	2022	US	1052	Gray-level co-occurrence matrix texture	Two-class BANN	AUC:0.75

US, ultrasonography; CEUS, contrast-enhanced ultrasonography; HRUS, high-resolution ultrasonography; RTE, real-time elastography; SWE, shear wave elastography; AUROC, area under ROC curve; Hist, histogram; GLCM, Gray-Level Co-occurrence Matrix; GLRLM, Gray-Level Run-Length Matrixes; NGLDM, Neighboring Gray-Level Dependence Matrix; FLBP, fuzzy local binary pattern; SVM, Support Vector Machines; PNN, Probabilistic Neural Network; KNN, k-Nearest Neighbor; ANN, artificial neural network; 1-NN, first–Nearest Neighbor; DT, Decision Tree; RT, Random Tree; RF, Random Forest; RBF-NN, radial basis function-neural network LDA, Linear Discriminant Analysis; GMM, Gaussian Mixture Model; QDA, Quadratic Discriminant Analysis; NB, Naive Bayes; FD, Fractal Dimension; LBP, Local Binary Pattern; FS, Fourier Spectrum; LTE, Laws Texture Energy; MLP, multi-layered perceptron; Two-class BANN, two-class Bayesian artificial neural networks; na, not available.

In 1989, Hirning et al. (47) published the first study to differentiate thyroid nodules based on ultrasound texture analysis. The overall accuracy of their classification system in classification has reached 85%. Chang et al. (49) used 78 texture features to describe thyroid ultrasound images and applied SVM to classify the images, achieving a remarkable accuracy rate of 100%. Acharya et al. (31, 54–57, 63) proposed a CAD system for automatically classifies malignant and benign thyroid nodules using 3D high-resolution and contrast-enhanced ultrasound (CEUS) images. The classification accuracy of different classifiers tested with these features ranged from 98.1% to 100%, indicating that the CAD system could support radiologists in identifying the nature of thyroid nodules. Raghavenra et al. (67) proposed a fusion method to identify the maturity of thyroid lesions, that is, the spatial gray scale correlation feature (SGLDF) and fractal texture system fusion. The classification efficiency of the SVM classifier was high, with an accuracy rate of 97.5% and a maximum AUROC of 0.95.To determine which ML classifiers have higher classification performance for thyroid nodules, Zhang et al. (69) nine ML classifiers (K-NN, CNN, Random Forest, Logistic, adaptive enhancement, Naive Bayes, neural networks, etc.) were evaluated for their classification performance of thyroid nodules using conventional ultrasound and real-time elastography features in 2064 thyroid gland samples, and compared to experienced radiologists. The Random Forest algorithm demonstrated the highest diagnostic performance among all the classifiers tested. Based on the handcrafted image features, Ouyang et al. (70)analyzed the classification efficacy of linear and nonlinear ML methods when processing thyroid data. They found that both methods had similar accuracy and a simpler prediction process compared to a CAD system, as there was no need to preprocess the image or extract texture features from it.

#### 3.3.2 DL

The integration of DL techniques with ultrasonography has garnered considerable interest in the identification of benign and malignant thyroid nodules (**Table 2**). Based on the DL model, it can learn useful texture features to extract features or classify thyroid nodules automatically in ultrasound images, which overcomes the limitations of manual methods.

**Table 2:** Main results of DL algorithm in thyroid nodules ultrasound image studies.

Reference	Published year	Number of subjects	Type of DL	Main Performance
**Ma et al** (76)	2017	15000 images	fusion of two pre-trained CNNs on the ImageNet	Accuracy:83.0%; Sensitivity: 82.4%; Specificity: 85.0%.
**Ma et al** (43)	2017	22123 images	CNN	Sensitivity: 91.5%
**Chi et al** (77)	2017	693 images	Fine-Tuning DCNN	Accuracy:99.1%
**Zhu et al** (78)	2017	298 nodules	Fine-Tuning DCNN (ResNet18-based network)	Accuracy:93.8%
**Gao et al** (79)	2018	342 nodules	multiple-scale CNN	Accuracy:82.2%
**Peng et al** (80)	2021	18049 images	ThyNet	AUROC:0.921.
**Zuo et al** (81)	2018	19260 images	Alexnet CNN	Accuracy:86.0%
**Zhu et al** (82)	2018	467 nodules	DNN	Accuracy:87.2%
**Buda et al** (83)	2019	1377 images (training set:1278; test set: 99)	Multitask DCNN	Sensitivity:87.0%; Specificity:52.0%.
**Guan et al** (84)	2019	2836 images (training set:2437; test set:399)	DL(Inception-v3)	Sensitivity: 93.3%; Specificity: 87.4%. AUROC:0.87
**Li et al** (85)	2019	42952 training samples and 2692 test samples	DCNN	Sensitivity: 93.4%; Accuracy:89.8%; AUROC:0.95
**Nguyen et al** (86)	2019	237 nodules	DCNN	Accuracy:90.88%
**Song et al** (87)	2019	1358 nodules Test set (internal:55; external:100).	DL(Inception-v3)	internal test set: Sensitivity:95.2%; external test set: Se:94.0%
**Song et al** (88)	2019	4309 images	multitask cascade CNN	Accuracy: 98.2%
**Sundar et al** (89)	2019	2525 training samples and 613 test samples	Inception-v3, VGG-16	Inception-v3+CNN: Accuracy:93.0%; VGG-16+CNN: Accuracy:79.0%;
**Wang et al** (90)	2019	5007 nodules	YOLOv2 neural network	Accuracy:90.3%
**Nguyen et al** (91)	2020	450 images	DCNN	Accuracy:92.1%
**Wang et al** (64)	2020	7803 images	CNN	Accuracy:84.6%
**Wu et al** (92)	2021	2082 images(90% training images and 10% testing images)	CNN(ResNet-50、Inception-Resnet v2、Desnet-121)	ResNet-50: Accuracy:87.4% Inception-Resnet v2: Accuracy:84.6% Desnet-121: Accuracy:84.6%
**Zhu et al** (93)	2021	16401 training samples and 1000 test samples	CNN (BETNET)	Accuracy:98.3%
**Liu et al** (94)	2021	163 pairs images	IF-JCNN	Accuracy:89.6%
**Kim et al** (95)	2022	12327 training samples and 3082 test samples	DL (VGG16, VGG19, ResNet)	VGG16: Accuracy:78.0% VGG19: Accuracy:74.0% ResNet: Accuracy:75.0%

Ma et al. (76) first proposed the classification of thyroid nodules based on the CNN fusion method. Their findings indicated an 83.02% diagnostic accuracy for this approach. Chi et al. (77) first attempted to combine the DL method with the TI-RADS scoring system to propose a classification system for thyroid images, utilizing the deep CNN GoogLeNet. By fine-tuning the existing DL network, they achieved a classification accuracy of 99.13%, leading to improved effectiveness of CAD systems in thyroid nodule evaluation. Peng et al. (80) developed a ThyNet-based DL model to differentiate benign and malignant thyroid nodules in a multicenter study, which showed that the AUROC was significantly higher in subjects with ThyNet diagnosed benign and malignant thyroid nodules than in radiologists (0.922 vs 0.839, P< 0.0001). And With the assistance of ThyNet, the number of fine needle aspirations decreased from 61.9% to 35.2%, while the number of missed malignant thyroid nodules decreased from 18.9% to 17.0%. It was concluded that ThyNet could significantly improve radiologists’ diagnosis and help to reduce unnecessary fine needle punctures of thyroid nodules.

Nguyen et al. (86) have developed a method for feature extraction from thyroid images using a cascade classifier architecture to enhance the performance of CAD systems for thyroid nodule classification. This approach combines both handcraft and DL, achieving an overall accuracy of 90.88%. Considering the differences in the DL network structure and the imbalance of image samples, the same group (91) artificially reduced the influence of the imbalance of training samplesby employing a weighted binary cross-entropy loss function in training multiple CNN models. This method achieved a 92.05% accuracy rate for thyroid ultrasound images. Wu et al. (92) combined ACR TI-RADS with CNN to train three commonly used DL algorithms to distinguish malignant from benign thyroid nodules in TI-RADS 4 and TI-RADS 5. The method showed a significant ability to distinguish malignant and benign nodules, demonstrating high clinical value. Liu et al. developed a joint convolutional neural network (IF-JCNN) based on information fusion to improve the diagnostic performance of thyroid nodules. The IF-JCNN was able to achieve an accuracy and AUROC of 0.896 and 0.956, respectively, which outperformed those obtained using only US images (94). Since multiple images from different angles are necessary for a thorough thyroid ultrasound examination, most methods only utilize a single US image for diagnosis. Wang et al. (96) proposed a new CNN structure for attention-based feature aggregation networks that can aggregate features extracted from multiple images in a single inspection. This method improves the ability to identify malignant thyroid nodules using different views.

#### 3.3.3 S-Detect

**Figure 2:**
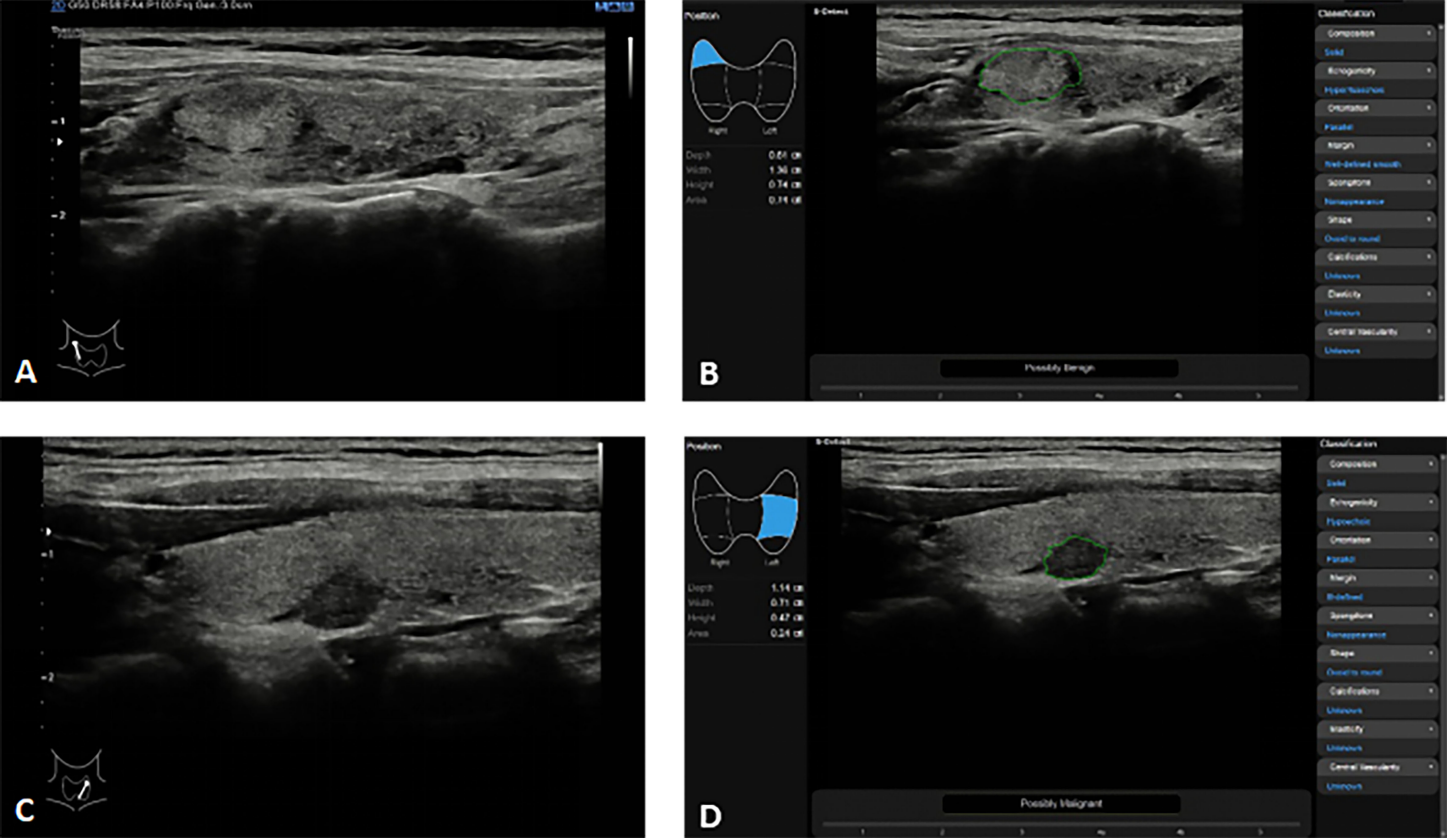
Thyroid nodules S-detect technique in the Samsung RS80A ultrasound system. (**A, B**) In a 35-year-old woman with right Hashimoto’s thyroiditis with focal fibrosis on conventional ultrasound **(A)**, S-Detect comes to the correct conclusion through analysis as “Possibly Benign” **(B)**; **(C, D)** In a 52-year-old woman with left thyroid cancer on conventional ultrasound **(C)**, S-Detect comes to the correct conclusion through analysis as “Possibly Malignant” **(D)**.

S-Detect (Samsung RS80A ultrasound system, Seoul, Korea) is the first commercially available ultrasound CAD based on DL technology for thyroid imaging. The system employs a CNN model that is trained using various TI-RADS hierarchical knowledge to automatically identify and analyze multiple grayscale ultrasound image features, including the internal structure, echo height, boundary, direction, and shape of thyroid nodules. After selecting the region of interest, the system can quickly determine whether the nodule is benign or malignant, either automatically or through manual intervention (**Figure 2**). Many studies have been carried out on the effectiveness of S-Detect system in differentiating between malignant and benign thyroid masses (97–109) (**Table 3**). Despite being a novel technique, its clinical applicability remains controversial, and different experiments have yielded varying results. Several studies have evaluated the diagnostic efficacy of the S-Detect system for identifying malignant thyroid nodules. Choi et al. (97) indicated that the difference in sensitivity of the S-Detect system for the diagnosis of malignant thyroid nodules compared to radiologists with 20 years of experience was not statistically significant (88. 4% *vs* 90.7%, *P* > 0.05), while the specificity(94.9% *vs* 74.9%, *P*<0.05) and discriminative power (AUROC 0.92 *vs* 0.83, *P*<0.05) of the S-Detect system were inferior to those of experienced radiologists. In contrast, the results of the S-Detect test by Gitto et al. (98) displayed no statistically significant difference in specificity between the software and radiologists with 5 years’ experience (66.7% *vs* 81.3%, *P* > 0.05)., but the diagnostic sensitivity of the software was inferior to the CAD system (21.4% *vs* 78.6%, P<0.05). Yoo et al. (109) found that the S-Detect system had comparable diagnostic performance to a radiologist with 10 years of experience, and could improve the diagnostic sensitivity and negative predictive value of less experienced radiologists. Jeong et al. (108) found that experienced radiologists had higher sensitivity and accuracy than less experienced radiologists when using the S-Detect system. A subgroup meta-analysis by Zhao et al. (107) showed that the S-Detect system had similar sensitivity to experienced radiologists, but lower specificity.

**Table 3:** Summary of related studies reported on thyroid nodules of compared diagnostic efficacy between the S-Detect and experienced radiologists.

Author	Nodules	Sensitivity		Specificity		Accuracy		PPV		NPV		AUROC	
	Benign/ maligant	S-D	Ra	S-D	Ra	S-D	Ra	S-D	Ra	S-D	Ra	S-D	Ra
**Choi et al** (97)	102 (59/43)	90.7%	88.4%	74.6%	94.9%	81.4%	92.2%	72.2%	92.7%	91.7%	91.8%	0.83	0.92
**Gitto et al** (98)	62 (48/14)	21.4%	78.6%	81.3%	66.7%	67.7%	69.4%	25.0%	40.7%	78.0%	91.4%	-*	-*
**Yoo et al** (109)	117 (67/50)	80.0%	84.0%	88.1%	95.5%	84.6%	90.6%	83.3%	93.3%	85.5%	88.9%	0.84	0.90
**Jeong et al** (108)	100 (56/44)	88.6%	84.1%	83.9%	96.4%	86.0%	91.0%	81.3%	94.9%	90.4%	88.5%	-*	-*
**Xia et al** (106)	180 (85/95)	90.5%	81.1%	41.2%	83.5%	67.2%	82.2%	63.2%	84.6%	79.5%	79.8%	-*	-*
**Kim et al** (99)	218 (132/86)	80.2%	84.9%	82.6%	96.2%	81.7%	91.7%	75.0%	93.6%	86.3%	90.7%	-*	-*
**Fresilli et al** (100)	107 (80/27)	70.4%	81.5%	87.5%	88.8%	65.5%	71.0%	89.7%	93.4%	-*	-*	0.79	0.85
**Wei et al** (101)	204(112/92)	91.3%	83.7%	65.2%	63.4%	77.0%	72.5%	68.3%	65.3%	90.1%	82.6%	0.78	0.74
**Han et al** (102)	454 (287/167)	97.6%	97.6%	21.6%	36.2%	49.6%	58.8%	42.0%	-*	93.9%	-*	-*	-*
**Chung et al** (103)	165 (140/25)	92.0%	72.0%	87.9%	85.0%	88.5%	83.0%	57.5%	46.2%	98.4%	94.4%	-*	-*
**Barczyński et al** (104)	50 (40/10)	90.0%	90.0%	95.0%	80.0%	94.0%	82.0%	81.8%	52.9%	97.4%	97.0%	-*	-*
**Molnár et al** (105)	200 (185/15)	88.6%	80.0%	88.1%	40.5%	88.0%	43.5%	-*	-*	-*	-*	0.94	0.66

PPV, Positive predictive value; NPV, negative predictive value; AUROC, area under the summary receiver operating characteristic curve; S-D, S-Detect; Ra, Radiologist. ∗The exact data were not reported in the original articles.

Xia et al. (106) were the first to utilize the S-Detect software to evaluate thyroid cancer subtypes. They found that the S-Detect system exhibited higher diagnostic sensitivity than experienced radiologists in detecting papillary thyroid cancer and follicular thyroid cancer. Nonetheless, the radiologists demonstrated superior diagnostic specificity compared to CAD systems. Kim et al. (99) evaluated the diagnostic efficacy of the S-Detect™ software (Rs85A) in its new version, which was assigned to classify calcification into four distinct categories. However, the accuracy of calcification identification limits the diagnostic performance of S-Detect. In terms of characterizing thyroid nodules, the S-Detect system and radiologists generally agreed on most sonographic features, but there were discrepancies when it came to margin definition. The study of Choi et al. (97) considered that radiologists and the S-Detect system described composition, orientation, echogenicity, and sponginess in substantial agreement (Kappa= 0.66, 0.74, 0.73, 0.66, respectively), while marginal definition showed a fair greement (Kappa = 0.239). Similar findings were reported by Xia et al (106). In addition, Gitto et al. (98) performed an inter-observer agreement between the S-Detect system and the radiologist with a Kappa value of only 0.03 for the margin assessment.

S-Detect is the first commercially available ultrasound CAD based on DL technology for Thyroid. It is specifically designed to support inexperienced radiologists in identifying thyroid nodules’ ultrasound characteristics and thereby enhance their diagnostic accuracy. However, despite its promising potential for clinical use, the S-Detect system’s performance is still largely dependent on the operator. Moreover, the system necessitates the manual input of certain features, and several attempts may be required to segment lesions correctly. Therefore, the system’s performance requires further development, including automatic detection of nodule calcifications and margins. This development will not only save analysis time but also enhance physicians’ overall performance in diagnosing nodules.

### 3.4 Prediction of lymph node metastasis in thyroid cancer

Assessing the recurrence and prognosis of thyroid cancer heavily relies on the status of lymph nodes, making it a significant indicator in the diagnostic process (110). Among various imaging techniques, ultrasound has become a preferred method due to its non-invasiveness, real-time monitoring, and convenience, providing essential information for diagnosis and treatment (111). To predict lymph node metastasis, the traditional risk prediction model based on risk factors such as tumor size, microcalcification, Hashimoto’s disease (112–115), and blood markers (116, 117), has been commonly used. Predictive models have been constructed to assess the lymph node status of thyroid cancer patients using ultrasound examination, with the area under the AUROC ranging from 0.67 to 0.80. At the same time, other analytical methods, especially radiomics and CNN models, have attracted significant attention because of their feasibility in exploring the correlation between ultrasonic features and the lymph node status of thyroid cancer (118, 119).

The CAD system utilizing DL algorithm was applied to predict lymph node status of thyroid cancer. Lee et al. (120) developed a CAD system using the VGG-Class activation map model to determine lymph node status. The study found that the model’s accuracy was 83.0% and exhibited good diagnostic performance. The system also provides reliability scores and identified regions associated with lymphatic metastasis from ultrasound images. Notably, this study established the first DL-based CAD system intended to assess lymph node status. Some scholars (120) developed a CAD system based on the CNN model to locate and identify the lymph node status of thyroid cancer. The results showed that the accuracy of the CAD system is 83.0% on the test set and effectively detected and diagnosed the location and nature of lymph nodes.

However, the application of the DL algorithm in auxiliary ultrasonic image diagnosis is not mature enough. One reason is the limited number of available ultrasonic images, which also have low resolution that prevents the algorithm from detecting typical features. Additionally, most current studies do not consider various clinical data, such as medical history, clinical inspection results, and other relevant information (121). Some scholars (122) proposed a deep multichannel learning network called MMC-Net to predict lymphatic metastasis of thyroid cancer. The study used clinical data, two-dimensional ultrasound and color Doppler flow imaging (CDFI) images as inputs and proposed a new index to compare the contribution of different channels to prediction. The proposed multi-channel DL network achieved an average F1 score of 0.888 and an average AUC of 0.973, outperforming three single-channel networks. These results indicate that the MMC-Net model is a more effective approach for predicting lymphatic metastasis of thyroid cancer. In recent years, researchers have shown interest in the application of radiology to predict the lymph node status of thyroid cancer. This has been achieved through the use of quantitative medical imaging features. By mining quantitative image feature data in a high-throughput manner and integrating it into clinical decision-making systems, it has been possible to improve the diagnostic accuracy of clinicians (119, 123, 124). Liu et al. (119) analyzed the lymph node status of patients with preoperative thyroid papillary carcinoma based on radiological methods. The radiomics method showed a prediction accuracy of 0.712, indicating the feasibility of radiological analysis of ultrasonic images of patients with thyroid papillary carcinoma. Jiang et al. (125) developed a multimodal ultrasound-based nomogram to predict lymph node metastatic status in papillary thyroid carcinoma. Multimodal ultrasound techniques include shear wave elastography and conventional ultrasound. The results showed that for univariate analysis, the radiological features of B-mode ultrasound and shear-wave elastography radiomics score were significantly correlated with lymph node status. However, the B-mode ultrasound radiomics score did not appear in the final nomogram.

Accurate identification and complete removal of metastatic lymph nodes during preoperative thyroid cancer treatment is crucial for preventing postoperative recurrence. The future CAD system holds promise in predicting metastatic lymph nodes with greater efficiency and accuracy, providing valuable insights for clinical diagnosis and treatment decision-making.

## 4 Challenges and future perspectives

In clinical ultrasound medicine, it is controversial when AI technology can be automatically applied in the clinic, with speculations for the time ranging from a few years to decades. Despite many studies that have confirmed the effectiveness of AI and achieved satisfactory results, most articles have used retrospective analysis or single-center controlled studies, which may lead to inevitable selection bias. For example, the samples in the training set are small, or the selected samples are not from the screening of thyroid nodules, but from the thyroid nodule population with pathological results, which leads to the unrepresentative model or the wrong description of the real population, thus affecting the universality of the model. Therefore, detailed system verification is needed before AI is applied to practical clinical practice. In order to carry out reliable and independent clinical verification, multi-center prospective research is needed in the future, and appropriate inclusion/exclusion criteria are set to make the selection of target population representative, and unused data sets are used for external verification. When the learning model adjusts itself too much on the training data set or the data set used for model development cannot fully represent the patient range (target population) to be applied to clinical practice, over-fitting or spectrum bias will occur (126, 127). Overfitting and spectrum bias may lead to overestimation of accuracy and generalization ability. Therefore, in order to correctly verify the accuracy of AI, doctors should evaluate the performance of AI by avoiding the influence of over-fitting and spectrum bias. DL can handle the complex relationship between dependent variables and independent variables and can make abstract inference at multiple levels. However, this complexity also makes the model a “black box” where the decision making mechanism is not clearly demonstrated, which is not conducive to building social acceptability (128), so further research is needed to address model interpretability or explainability.

The downside of the AI tool is that it can’t solve multiple tasks, and being good at one task doesn’t necessarily mean being good at other tasks. In addition, the difference between the actual efficiency of AI results and the expected results and the cost-effectiveness must be proved by complex and extensive investigations. In the current medical environment, the acquisition of reasonable regulations and reimbursement policies from relevant departments is crucial for the progress of AI technology. At present, a common shortage of AI tools is that they cannot resolve multiple tasks. There is currently no comprehensive AI system capable of detecting multiple abnormalities throughout the human body.

We believe that the future AI system will increase the efficacy of detecting thyroid nodules and predicting the lymph node status of thyroid cancer. At the same time, it can further distinguish specific benign and malignant diseases, such as thyroiditis, thyroid adenoma and nodular goiter. Besides, In the aspect of thyroid ultrasound-guided puncture biopsy and microwave ablation, the AI navigation intervention system can be further developed and perfected. The puncture position can be monitored in real-time by computer software to improve the accuracy of puncture. In addition, complementary information is provided by creating a DL model trained on multimodal images to further improve the diagnostic performance of the DL model.

## 5 Conclusion

At present, the application of AI in medicine has achieved satisfactory achievement, especially in the recognition and diagnosis of imaging pictures. It is rapidly emerging as a promising adjunct to thyroid ultrasound imaging tasks, satisfying the desire of clinical care to improve the efficiency of medical imaging. As an advanced technology, AI has changed the dependence and subjectivity of traditional ultrasound diagnosis on operator’s experience. In addition, AI can also improve diagnostic efficiency and reduce the burden on radiologists. With the continuous increase in the amount of data, AI will be the domain development direction of thyroid ultrasound diagnosis in the future. In order to utilize AI wisely, radiologists must keep up to date with its feasibility, consider the strengths and limitations of different algorithms, understand the impact of overfitting and spectral bias on AI performance, understand that DL technology has its own “black box” nature (lack of interpretability or explainability), and that radiologists need to attempt to compensate for its shortcomings by building rich heterogeneous image datasets, using unused datasets for external validation, etc. We believe that AI will not replace the dominant role of human doctors. Still, AI can provide a credible rationale for doctors to make clinical decisions in some regions of imaging functions.

## Author contributions

C-LC, Q-LL, and JL contributed to the conception and design of the study. JC and JT searched and reviewed studies, extracted and analyzed the data, and wrote the first draft of the manuscript. L-NS, W-XL, and YX reviewed and edited the manuscript. JC, T-TD, X-WC, and JL directed the project and contributed to discussion as well as reviewed and edited the manuscript. All authors contributed to the article and approved the submitted version.
